# Impact of Clinical Factors on Bone Microarchitecture in Patients with Turner Syndrome

**DOI:** 10.2174/0118715303434041251216094435

**Published:** 2026-04-29

**Authors:** Isabella Nardone, Sium Wolde Sellasie, Simona Zaccaria, Marina Passeri, Luca Giudice, Lorenza Mattia, Cristina Giusto, Rossella Antonelli, Stefania Falcone, Laura Giurato, Luigi Uccioli

**Affiliations:** 1 Division of Endocrinology and Diabetes, Department of Surgical Sciences, CTO Andrea Alesini Hospital, University of Rome Tor Vergata, 00133, Rome, Italy;; 2 PhD School of Applied Medical-Surgical Sciences, University of Rome Tor Vergata, 00133, Rome, Italy;; 3 Division of Endocrinology and Diabetes, Fondazione Policlinico Tor Vergata, Rome, Italy;; 4 Department of Experimental Medicine, Sapienza University of Rome, Rome, Italy

**Keywords:** Turner syndrome, bone microarchitecture, trabecular bone score, estrogen replacement therapy, bone fragility, congenital disease

## Abstract

**Introduction:**

Patients affected by Turner syndrome (TS) suffer from bone fragility and a high risk of fractures. The health of bone microarchitecture has proven to be a very important element in reducing the risk of fractures. The present study aimed to describe bone characteristics of patients with TS and highlight clinical factors that can influence bone microarchitecture, evaluated by the trabecular bone score (TBS).

**Methods:**

This single-centre retrospective study included 41 patients with TS who attended the Endocrinology Day Hospital of the CTO Hospital of Rome between 2016 and 2024. Anthropometric, clinical, and densitometric data were collected and analysed using unadjusted univariate models, age- and body mass index-adjusted models, and multivariate analysis to investigate their effects on TBS.

**Results:**

There was a positive association between TBS and duration of estrogen replacement therapy (ERT). Patients who were constantly treated with ERT showed higher bone mineral density (BMD) at the lumbar spine and femoral neck (*p*=0.026 and *p*=0.040, respectively) and TBS (*p*=0.031). The presence of metabolic diseases appeared to have a negative influence on TBS (*p*=0.034).

**Discussion:**

Regular ERT and longer treatment duration appear to be the most important factors to significantly improve BMD and TBS levels in patients with TS. On the contrary, diagnosis of metabolic diseases significantly affect bone microarchitecture.

**Conclusion:**

Healthcare providers should pay particular attention to timely ERT and the prevention of metabolic complications, since these are the main factors that appear to improve bone architecture in this specific population.

## 1 INTRODUCTION

Turner syndrome (TS) is a rare congenital disease, genetically determined by partial or complete deletion of one of the two X chromosomes [1-3]. The phenotype varies considerably from case to case, depending on whether patients with complete deletion or mosaicism are considered [4-7]. The peculiar characteristics of TS are short stature, typical facial and neck features, heart [8], kidney, and ear involvement [9], predisposition to develop autoimmune and metabolic diseases, and bone fragility [4, 9-14]. Early diagnosis of TS is crucial because timely estrogen replacement therapy (ERT) and growth hormone therapy (GHT) during childhood allow improved stature growth and good estrogenization [6, 7]. Furthermore, peak bone mass can be reached, and musculoskeletal and metabolic damage can be prevented [4, 15-17]. The risk of bone fracture is 1.4-2.2 times higher in patients with TS than in the healthy population, especially in the forearms, metacarpal site, proximal hip, and lumbar spine [18, 19]. Fracture risk in these patients is high since childhood, and it tends to increase with age [19, 20]. Although there is a genetic influence, among the factors that affect bone health, the most important is the lack of estrogens [21, 22]. Patients who are not properly treated with estrogens present a predisposition to bone fractures, which increases to 45% [23]. These hormones play a critical role in bone remodelling and in maintaining bone mineral density (BMD). Estrogens interact with their estrogen receptors (ERs), which are expressed throughout the body, especially in bone tissue. Estrogens bind to their ER and induce the formation of transcription factors in the cellular nucleus to coregulate gene expression [24]. Estrogen deficiency has been shown to increase apoptosis of osteocytes and osteoblasts, while decreasing osteoclast apoptosis and promoting their differentiation through the RANK-RANKL signaling pathway [25-28]. Beyond regulating BMD through osteoclast and osteoblast activity, estrogens also appear to inhibit sclerostin and enhance sensitivity to mechanical loading, thereby contributing significantly to healthy bone mineralization and microarchitecture, particularly during growth [29-32]. In addition to reductions in BMD, bone microarchitecture and geometry can be impaired, with vertebrae and femurs being smaller and cortical thickness thinner compared to the control population [18]. The risk of fracture may also be influenced by autoimmune and metabolic comorbidities associated with TS. Hearing impairment may result in a greater predisposition to falls [12]. GHT has an effect on stature prognosis and other clinical presentations of TS, but its impact on the areal BMD and bone microarchitecture is still controversial [13, 14]. The gold standard method for studying BMD and fracture risk is dual-energy X-ray absorptiometry (DXA), performed on the lumbar spine and the proximal hip [33-36]. Low BMD is considered one of the main causes of bone fragility. However, there is evidence that the BMD of patients with TS, evaluated by DXA, is underestimated due to their short stature and reduced bone size [18, 37]. Trabecular bone score (TBS) is another interesting parameter that can be obtained from the lumbar scan performed by DXA [38]. Based on a grey scale, TBS provides indirect information on the integrity of the bone trabecular microarchitecture. When degraded, TBS represents an independent risk factor for fragility fractures [39]. TBS does not appear to be affected by short stature and small bone size; therefore, it could be a useful tool in bone health and fracture risk assessment in patients with TS. Several clinical factors have been shown to have a significant influence on bone microarchitecture [40]. The present study aimed to describe bone characteristics of patients with TS and highlight clinical factors that can influence bone microarchitecture evaluated by TBS.

## 2 MATERIALS AND METHODS

This single-centre retrospective study involved 41 female patients affected by TS, attending the endocrinology day hospital of the CTO Hospital of Rome between 2016 and 2024 for clinical management and screening of complications. All included patients have been evaluated by DXA according to medical prescription to investigate bone fragility. Informed consent was obtained from all participants included in the study. Inclusion criteria were as follows: TS diagnosis with karyotype analysis and age ≥ 18 years old. Exclusion criteria were previous or concurrent treatment that might influence bone health (antiresorptive drugs and corticosteroids).

### 2.1 Data Collection

Data were collected from medical records and included the following variables: age (years), height (m), weight (kg), body mass index (BMI, kg/m^2^), and karyotype, classified as classic 45,X or non-classic (mosaic 45,X/46,XX; mosaic 45,X/46,XY; isochromosome Xq; and more complex karyotypes, including X–autosome translocations, 45X/47XXX mosaicism, and partial deletions). Information on estrogen replacement therapy (ERT) was recorded, including regularity (defined as continuous intake without interruptions longer than three months), duration (years), age at initiation (years), and formulation (oral or transdermal). Growth hormone therapy (GHT) and its duration (years) were also documented. Additional clinical variables included the presence of autoimmune diseases (*e.g.*, autoimmune thyroiditis, vitiligo, celiac disease), anatomical malformations (*e.g.*, kidney or heart malformations, hearing impairment), metabolic disorders (*e.g.*, type 2 diabetes mellitus, arterial hypertension, dyslipidemia), smoking habits, and history of fractures. Bone health measures included bone mineral density (BMD, g/cm^2^) at the lumbar spine, femoral neck, and total hip assessed by DXA, as well as trabecular bone score (TBS).

### 2.2 Evaluation of BMD

 BMD was evaluated by an expert operator of the CTO Hospital Bone Unit using a Hologic Discovery A densitometer. The following sites were examined: lumbar spine (L1-L4), femoral neck, and total hip. A Z score < -2.0 SD was considered diagnostic of low bone mass. The TBS iNsight (Version 3.0.2.0, Medimaps, Geneva, Switzerland) was used to process the data relating to the trabecular microarchitecture starting from the lumbar scan obtained by DXA. TBS values greater than 1.350 were considered normal, values between 1.350 and 1.200 were considered partially degraded, and values lower than 1.200 were considered highly degraded [38]. Phantom scans were performed daily for quality control.

### 2.3 Statistical Analysis

Statistical analysis was performed using Jamovi software version 2.3.28 [41]. Quantitative variables were expressed as mean ± standard deviation (SD), Student's t-test was applied, and qualitative data were expressed as frequencies. BMD and TBS were compared, dividing patients according to regular ERT and the presence of classic or non-classic karyotype. A *p* < 0.05 was considered statistically significant. To explore the effect of clinical data (such as BMI, ERT duration, GHT duration, and the presence of autoimmune and metabolic complications and anatomical malformations) on TBS, linear regression analysis was conducted with an unadjusted model and, subsequently, with age- and BMI-adjusted models. Subsequently, a multivariate regression analysis was performed, including metabolic diseases and the duration of ERT. Our multiple comparisons were tested using Bonferroni correction (*p* < 0.006).

## 3 RESULTS

A total of 41 patients affected by TS, with a mean age of 38 ± 8.77 years, were included in the study. The mean BMI was 26.3 ± 5.16 SD kg/m^2^; the mean BMD was 0.886 ± 0.123 SD g/cm^2^ in the lumbar spine, 0.698 ± 0.088 SD g/cm^2^ in the femoral neck, and 0.830 ± 0.102 SD g/cm^2^ in the total hip, respectively. Twenty-seven (65.9%) patients had normal BMD levels, and 14 (34.1%) patients had low bone mass (Z score <-2.0 SD). Mean TBS values ​​were 1.320 ± 0.101 SD; 18 patients had normal TBS values, 14 had degraded values, and 8 had severely degraded values. Most of the patients (n.28 - 68.3%) were constantly treated with ERT with a median therapy duration of 22 ± 17.5 SD years. The mean age at the start of ERT was 18.2 ± 4.77 SD years. Sixteen patients were treated with transdermal estradiol and 12 patients with oral estradiol formulation. The remaining 13 (31.7%) patients did not follow regular ERT due to contraindications or scarce adherence. About 82.9% of the patients received GHT during childhood/adolescence, at a dose of 0.44 mg/day, with a mean duration of 7.40 ± 3.40 years. No patient reported a history of spontaneous or traumatic bone fractures. Regarding the complications of the disease, 13 (31.7%) patients had cardiac malformations, 5 (12.2%) patients had kidney malformations, and 31 (75.6%) had autoimmune diseases. Other illnesses, such as arterial hypertension, T2DM, hepatic steatosis, and dyslipidaemia, were reported, respectively, by 5 (12.2%), 4 (9.7%), 16 (39%), and 6 (14.6%) patients. Only 5 (12.2%) patients reported the habit of smoking. The clinical and densitometric parameters are summarised in Table **1**.

Clinical, anthropometric, and densitometric parameters were analysed using a univariate unadjusted model and univariate age- and BMI-adjusted models (Table **2**).

A positive association was found between TBS and longer duration of ERT (*p* = 0.017), with greater statistical significance in models adjusted for age and BMI (*p* = 0.006; *p* = 0.007). A positive correlation was also found between TBS and BMD levels in the lumbar spine (*p* < 0.001) and femoral neck (*p*=0.044) in adjusted and unadjusted models. Higher levels of BMI were negatively associated with TBS (*p* = 0.05). No significant influence of GHT duration on TBS values was shown. The diagnosis of metabolic diseases showed significantly negative effects on TBS (*p* = 0.034), also in models adjusted for BMI (*p* = 0.034). No significant association was found with smoking habit, diagnosis of autoimmune diseases, or anatomical malformations. Statistically significant results were confirmed in a multivariate analysis, including metabolic diseases and the duration of ERT (Table **3**). After Bonferroni correction, a significant positive correlation was observed between ERT duration and lumbar spine BMD in the age-adjusted model.

None of the 41 patients had a history of bone fractures; therefore, it was not possible to perform an analysis of the ability of TBS to predict fracture risk.

Densitometric values were compared by dividing patients according to the presence of regular ERT (Table **4**).

**Fig. (1):**
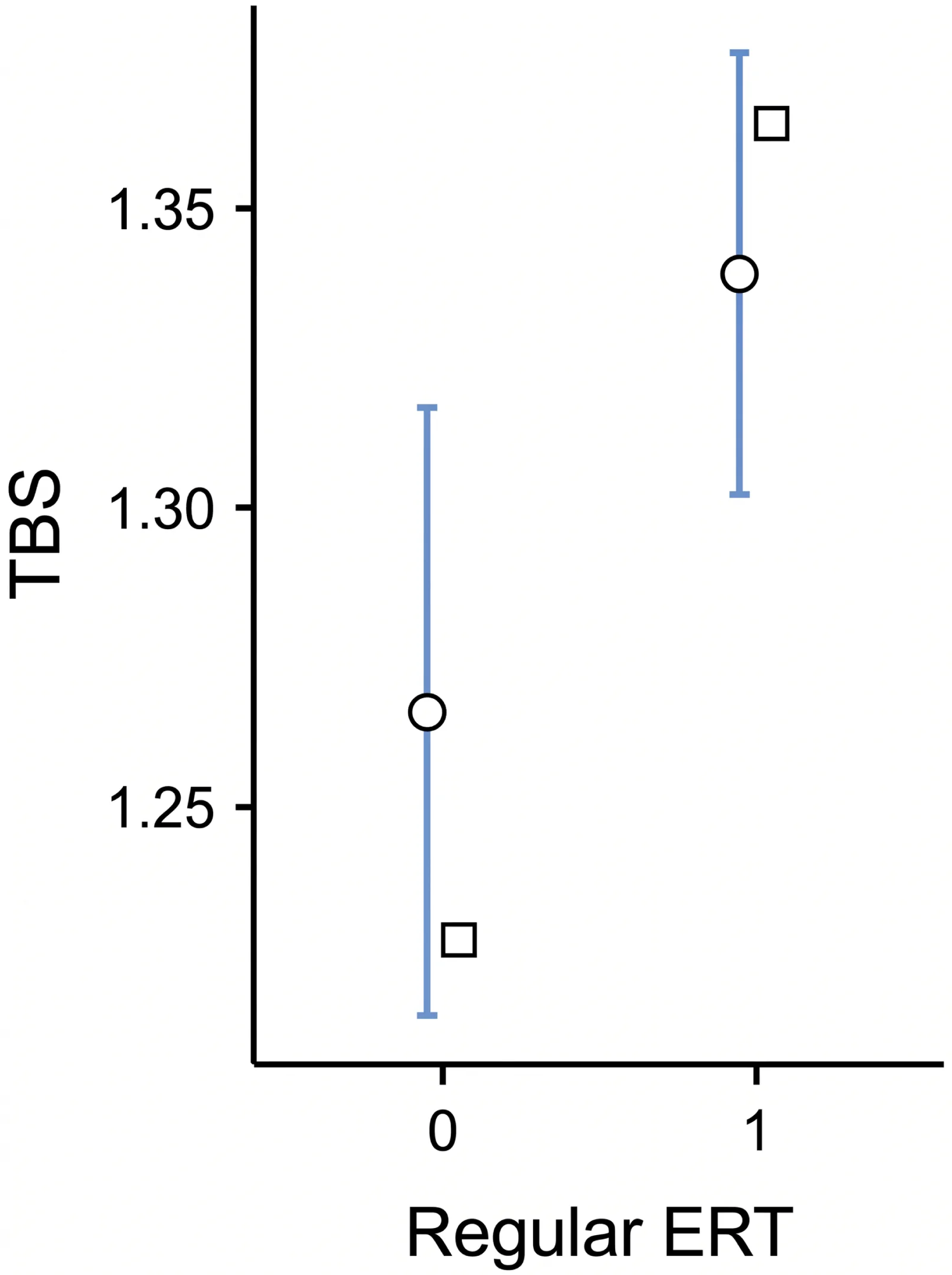
The patients following regular ERT showed higher levels of TBS (0=0.031).

The 28 patients who were constantly treated with ERT showed significantly higher levels of TBS (*p*=0.031) (Fig. **1**) and BMD in the lumbar spine and femoral neck, respectively (*p* = 0.026 and *p* = 0.040).

No significant differences in BMD or TBS were observed when patients were stratified according to classic *versus* non-classic karyotypes. The TS classic karyotype showed a longer mean duration of ERT, but without statistical significance (Table **5**).

## 4 DISCUSSION

TS is one of the most common chromosomal alterations; early diagnosis and timely replacement therapy are crucial for the prevention of complications [22]. In particular, bone fragility, which has serious consequences for general health and quality of life, can be prevented by initiating appropriate replacement therapy at the right time [18]. Beyond BMD depletion, bone microarchitecture impairment has been shown to significantly increase bone fragility. Taking into account the great phenotypic variability of patients with TS, this study aimed to evaluate the clinical factors that can influence bone microarchitecture.

In this specific population, previous literature demonstrates that the presence of adequate ERT and the duration of treatment improve bone health, restoring the risk of fracture to the levels of the general population [22, 42, 43]. A recent review investigated how timely and adequate ERT in patients with TS-related primary hypogonadism may improve bone outcomes, considering BMD, TBS, and fracture risk [44]. Another longitudinal study investigated BMD, TBS, and fracture risk in TS patients [43]. It was found that TBS was negatively correlated with age and with delayed ERT initiation; in addition, low levels of TBS were predictive of fractures.

Our results confirmed that patients regularly treated with ERT show significantly higher levels of BMD in the lumbar spine and femoral neck and better results in TBS. The duration of replacement therapy is positively correlated with TBS values. This result supports previous literature indicating that timely replacement of gonadal function allows the achievement of a good level of bone mass even in the presence of primary hypogonadism. Another study by Saito *et al*. investigated the effects of age at ERT initiation on bone quality in Japanese patients with TS [15]. They showed that early initiation of ERT may have positive effects on bone quality evaluated by TBS in this population. Due to the negative correlation observed among TBS, BMI [40, 45], and age, we performed a further analysis using models adjusted for BMI and age, which confirmed statistical significance [40].

As expected, in our study, a negative correlation was found between BMI and TBS levels. Higher BMI can be an obstacle to the accurate determination of BMD and TBS during a DXA lumbar scan; in fact, the amount of local soft tissue can lead to X-ray attenuation and image noise [46, 47]. Therefore, this parameter should be taken into account when evaluating bone microarchitecture by TBS.

Beyond BMI and technical limitations, obesity is related to systemic complications and significant inflammation of the tissues [48], including bone. Despite higher BMD values, a deleterious effect on bone health was detected in patients with obesity, along with reduced bone turnover and increased bone marrow adiposity. Therefore, deterioration of bone microarchitecture and bone fragility may persist [49, 50].

Data from previous literature and our study suggest that estrogen exposure is among the most important factors for bone microarchitecture health. It should be highlighted that, beyond bone, estrogens are also important for metabolic health. Women, after menopause, are more predisposed to develop metabolic diseases, such as dyslipidaemia and type 2 diabetes mellitus, with an increased risk of cardiovascular events [51]. In the case of TS patients, the lack of adequate estrogen exposure may predispose them to this phenomenon [52, 53]. In the studied population, the diagnosis of metabolic diseases negatively affected TBS levels. Although the findings of this study have been limited by the small sample size, they have been consistent with reports in the literature for the general population affected by metabolic conditions, particularly type 2 diabetes mellitus [54-59]. Particular attention should be paid to TS patients with metabolic alterations when evaluating bone health. More specific and longitudinal studies are needed to better characterize the association between metabolic health and bone fragility in this specific population.

As already highlighted in a recent meta-analysis, GHT has no direct effect on improving bone density and microarchitecture [34]. However, it is necessary to remember that GHT can provide benefits for metabolic health, which could affect the trabecular microarchitecture, as found in this study.

Patients affected by TS exhibit great phenotypic variability, especially in the presence of different karyotypic forms. The phenotype of patients with the classic 45,X form appears to be associated with more serious clinical characteristics, including anatomical malformations, more severe stature deficit, and bone fragility [60]. Very often, the typical phenotype shown by the classic form of TS allows for an earlier diagnosis and timely ERT, which has been shown to be among the most important factors in preventing bone complications. Even if statistical significance was not reached, in this study, a non-classic karyotype was associated with a shorter ERT duration and lower levels of TBS. Future studies may investigate TBS patterns in different TS karyotypes.

This study was limited by its retrospective design and small sample size, despite the rarity of the disease. No patients involved had a history of bone fragility; therefore, the study could not investigate fracture outcome. Furthermore, the data on ERT adherence were self-reported by patients, and reporting bias could not be excluded.

## CONCLUSION

TS is a complex and rare disease that requires comprehensive evaluation and management in specialized medical centres. Healthcare providers should pay particular attention to ERT adherence and prevention of metabolic complications, as these are the main factors that seem to improve the health of bone microarchitecture in this specific population. Larger longitudinal studies are required to investigate the effects of ERT on bone microarchitecture and reduction in fracture risk, and better characterize the effect of metabolic complications on bone health in TS patients.

## Figures and Tables

**Table 1:** Clinical and densitometric parameters of the total population.

**Variables**	**Total Population (n=41)**
Classic TS karyotype	13 (31.7)
Age (years)	38 ± 8.77
Height (m)	1.50 ± 0.05
Weight (kg)	59 ± 11.3
BMI (kg/m^2^)	26.3 ± 5.16
Regular ERT	28 (68.3)
ERT duration (years)	22 ± 17.5
Age at ERT initiation (years)	18.2 ± 4.77
Formulations of ERT	Transdermal 16 (57.2) Oral 12 (42.8)
GHT	34 (82.9)
GHT duration (years)	7.40 ± 3.40
Smoking habit	5 (12.2)
History of fractures	0 (0)
Metabolic diseases	23 (56.1)
• Type 2 diabetes mellitus	4 (9.7)
• Arterial hypertension	5 (12.2)
• Dyslipidaemia	6 (14.6)
• Hepatic steatosis	16 (39)
Autoimmune diseases	31 (75.6)
• Autoimmune thyroiditis	29 (70.7)
• Vitiligo	1 (2.4)
• Celiac disease	2 (4.87)
Anatomical malformations	20 (48.7)
• Heart malformations	13 (31.7)
• Kidney malformations	5 (12.2)
• Hearing impairment	8 (19.5)
BMD lumbar spine (g/cm^2^)	0.886 ± 0.123
BMD femoral neck (g/cm^2^)	0.698 ± 0.088
BMD total hip (g/cm^2^)	0.830 ± 0.102
TBS	1.320 ± 0.101
Low bone mass	14 (34.1)
Normal TBS	18 (43.9)
Partially degraded TBS	14 (34.1)
Degraded TBS	8 (19.5)

**Note:** Catgorical variables are presented as n (%) and continuous variables as mean ± standard deviation. **Abbreviations:** Turner syndrome (TS), body mass index (BMI), bone mineral density (BMD), trabecular bone score (TBS), growth hormone replacement therapy (GHT), estrogen replacement therapy (ERT).

**Table 2:** Influence of clinical, anthropometric, and densitometric parameters on TBS, analysed using an unadjusted univariate model and age- and BMI-adjusted univariate models.

-	**Unadjusted**			**Age-adjusted Model**			**BMI-adjusted Model**		
**Parameters**	**R^2^**	**t**	***p*-value**	**R^2^**	**t**	***p*-value**	**R^2^**	**t**	***p*-value**
TBS	-	-	-	-	-	-	-	-	-
BMI, kg/m^2^	0.092	-1.96	0.05	0.083	-1.86	0.07	-	-	**-**
ERT duration, years	0.207	2.56	**0.017**	0.266	3.01	**0.006**	0.255	2.93	**0.007**
GHT duration, years	0.012	-0.633	0.531	0.004	-0.37	0.714	0.012	-0.645	0.524
Metabolic diseases	-	-2.20	**0.034**	-	-1.82	0.076	-	-2.20	**0.034**
Autoimmune diseases	-	-1.71	0.095	-	-1.58	0.122	-	-1.94	0.06
Anatomical malformations	-	0.352	0.727	-	0.304	0.763	-	0.232	0.817
BMD lumbar spine, g/cm^2^	0.290	3.94	**<0.001**	0.291	3.95	**<0.001**	0.302	4.05	**<0.001**
BMD femoral neck, g/cm^2^	0.102	2.08	**0.044**	0.107	2.13	**0.04**	0.104	2.10	**0.043**

**Note:** Boldface indicates statistically significant *P* values. R2 was reported only for continuous variables.

**Table 3:** Multivariate regression of ERT duration and metabolic diseases.

-	R2	t	*p*
ERT duration	0.405	3.43	**0.002**
Metabolic diseases	-	-2.82	**0.009**

**Note:** Boldface indicates statistically significant *P* values. R2 was reported only for continuous variables.

**Table 4:** Descriptive and comparative analysis of patients divided by the presence of regular and non-regular ERT.

**Variables**	**Total Population (n=41)**	**Regular ERT (n=28)**	**Non-regular ERT (n=13)**	***p*-value**	**Effect Size**
**BMD lumbar spine (g/cm^2^)**	0.886 ± 0.123	0.915 ± 0.114	0.824 ± 0.122	**0.026**	-0.776
**BMD femoral neck (g/cm^2^)**	0.698 ± 0.088	0.708 ± 0.0788	0.648 ± 0.0957	**0.040**	-0.714
**BMD total hip (g/cm^2^)**	0.830 ± 0.102	0.845 ± 0.0965	0.797 ± 0.109	0.161	-0.480
**TBS**	1.320 ± 0.101	1.340 ± 0.0976	1.270 ± 0.0932	**0.031**	-0.758

**Note:** Boldface indicates statistically significant P values. Variables are presented as mean ± standard deviation.

**Table 5:** Descriptive and comparative analysis of patients divided by the presence of classic and non-classic karyotypes.

**Variables**	**Total Population (n=41)**	**Classic Karyotype 45,X (n=13)**	**Non-classic Karyotype (n=28)**	***p*-value**
**BMD lumbar spine (g/cm^2^)**	0.886 ± 0.123	0.866 ± 0.109	0.885 ± 0.189	0.947
**BMD femoral neck (g/cm^2^)**	0.698 ± 0.088	0.692 ± 0.108	0.679 ± 0.127	0.487
**BMD total hip (g/cm^2^)**	0.830 ± 0.102	0.826 ± 0.132	0.817 ± 0.092	0.946
**TBS**	1.320 ± 0.101	1.370 ± 0.076	1.310 ± 0.183	0.108
**ERT duration (years)**	22 ± 17.5	25 ± 7.35	17.8 ± 10.1	0.098

**Note:** Boldface indicates statistically significant *P* values. Variables are presented as mean ± standard deviation.

## Data Availability

The data will be available from the corresponding author upon reasonable request.

## LIST OF ABBREVIATIONS

TS: Turner Syndrome
ERT: Estrogen Replacement Therapy
GHT: Growth Hormone Therapy
T2DM: Type 2 Diabetes Mellitus
BMD: Bone Mineral Density
TBS: Trabecular Bone Score
DXA: Dual Energy X-Ray Absorptiometry
BMI: Body Mass Index
ER: Estrogen Receptor

## STANDARDS OF REPORTING

STROBE guidelines and methodology were followed.

## References

[1] Gravholt C.H., Viuff M., Just J., Sandahl K., Brun S., van der Velden J., Andersen N.H., Skakkebaek A. (2023). The changing face of turner syndrome.. Endocr. Rev..

[2] Wan K.L., Brown E.L., Krishnaswamy R., Kaub P.A. (2025). Turner syndrome.. J. Paediatr. Child Health.

[3] Tanoshima M., Tanoshima R., Takase H., Yamamoto D., Aoki S., Sakakibara H., Miyagi E. (2025). Karyotype and phenotype association in Turner syndrome with non-mosaic X chromosome structural rearrangements: Systematic review.. Congenit. Anom. (Kyoto).

[4] Gravholt C.H., Viuff M.H., Brun S., Stochholm K., Andersen N.H. (2019). Turner syndrome: Mechanisms and management.. Nat. Rev. Endocrinol..

[5] Wolff D.J., Van Dyke D.L., Powell C.M., Working Group of the ACMG Laboratory Quality Assurance Committee (2010). Laboratory guideline for Turner syndrome.. Genet. Med..

[6] Ibarra-Ramírez M., Campos-Acevedo L.D., Martínez de Villarreal L.E. (2023). Chromosomal abnormalities of interest in turner syndrome: An update.. J. Pediatr. Genet..

[7] Shankar K.R., Gravholt C.H., Backeljauw P.F. (2025). Evolution of health care in turner syndrome.. Am. J. Med. Genet. C. Semin. Med. Genet..

[8] de Groote K., Patel S.R., Mortensen K.H., Witvrouwen I., Duijnhouwer A., Brown N.M., Grewal J., Chatfield K.C., Dorfman A.T., Prakash S.K. (2025). Management of cardiovascular health issues in turner syndrome: expert insights and expanded recommendations from the 2024 guideline development team.. Am. J. Med. Genet. C. Semin. Med. Genet..

[9] Migliorelli A., Ciorba A., Manuelli M., Corazzi V., Stomeo F., Bianchini C., Pelucchi S., Monzani D., Genovese E., Palma S. (2025). Hearing loss and turner syndrome: A scoping review.. J. Int. Adv. Otol..

[10] Yoon S.H., Kim G.Y., Choi G.T., Do J.T. (2023). Organ abnormalities caused by turner syndrome.. Cells.

[11] Faienza M.F., Ventura A., Colucci S., Cavallo L., Grano M., Brunetti G. (2016). Bone fragility in turner syndrome: Mechanisms and prevention strategies.. Front. Endocrinol. (Lausanne).

[12] Khan N., Farooqui A., Ishrat R. (2024). Turner Syndrome where are we?. Orphanet J. Rare Dis..

[13] Lacka K., Pempera N., Główka A., Mariowska A., Miedziaszczyk M. (2024). Turner syndrome and the thyroid function: A systematic and critical review.. Int. J. Mol. Sci..

[14] Turner H.E., Johannsen E.B., Smyth A., Orchard E., Gravholt C.H. (2026). Approach to the patient with turner syndrome.. J. Clin. Endocrinol. Metab..

[15] Saito S., Koga E., Okada Y., Tsuburai T., Yoshikata H., Miyagi E., Sakakibara H. (2021). Effects of age at estrogen replacement therapy initiation on trabecular bone score in Japanese adults with Turner syndrome.. Osteoporos. Int..

[16] Kanakatti Shankar R., Quigley C.A., Isojima T., Mauras N., Chernausek S.D., Wasniewska M., Sas T.C.J. (2025). Growth and growth-promoting treatments in turner syndrome.. Am. J. Med. Genet. C. Semin. Med. Genet..

[17] Cleemann L., Holm K., Kobbernagel H., Kristensen B., Skouby O., Kryger Jensen A. (2017). Dosage of estradiol, bone and body composition in Turner syndrome: A 5-year randomized controlled clinical trial.. Eur. J. Endocrinol..

[18] Ikegawa K., Hasegawa Y. (2022). Fracture risk, underlying pathophysiology, and bone quality assessment in patients with Turner syndrome.. Front. Endocrinol. (Lausanne).

[19] Gravholt C.H., Juul S., Naeraa R.W., Hansen J. (1998). Morbidity in Turner syndrome.. J. Clin. Epidemiol..

[20] Attard C.C., Cameron-Pimblett A., Puri D., Elliot J., Wilson J.C., Talaulikar V.S., Davies M.C., Conway G.S. (2019). Fracture rate in women with oestrogen deficiency – Comparison of Turner syndrome and premature ovarian insufficiency.. Clin. Endocrinol. (Oxf.).

[21] Chiarito M., Piacente L., Chaoul N., Pontrelli P., D’Amato G., Grandone A., Russo G., Street M.E., Wasniewska M.G., Brunetti G., Faienza M.F. (2022). Role of Wnt-signaling inhibitors DKK-1 and sclerostin in bone fragility associated with Turner syndrome.. J. Endocrinol. Invest..

[22] Cameron-Pimblett A., Davies M.C., Burt E., Talaulikar V.S., La Rosa C., King T.F.J., Conway G.S. (2019). Effects of estrogen therapies on outcomes in turner syndrome: Assessment of induction of puberty and adult estrogen use.. J. Clin. Endocrinol. Metab..

[23] Lacka K. (2005). Turner’s syndrome--correlation between karyotype and phenotype.. Endokrynol. Pol..

[24] Shi V., Morgan E.F. (2024). Estrogen and estrogen receptors mediate the mechanobiology of bone disease and repair.. Bone.

[25] Stokes G., Herath M., Samad N., Trinh A., Milat F. (2025). ‘Bone health—across a woman’s lifespan’.. Clin. Endocrinol. (Oxf.).

[26] Hsu S.H., Chen L.R., Chen K.H. (2024). Primary osteoporosis induced by androgen and estrogen deficiency: The molecular and cellular perspective on pathophysiological mechanisms and treatments.. Int. J. Mol. Sci..

[27] Eriksen E.F. (2010). Cellular mechanisms of bone remodeling.. Rev. Endocr. Metab. Disord..

[28] Boyce B.F., Xing L. (2008). Functions of RANKL/RANK/OPG in bone modeling and remodeling.. Arch. Biochem. Biophys..

[29] Khosla S., Monroe D.G. (2018). Regulation of bone metabolism by sex steroids.. Cold Spring Harb. Perspect. Med..

[30] Sowińska-Przepiera E., Krzyścin M., Syrenicz I., Ćwiertnia A., Orlińska A., Ćwiek D., Branecka-Woźniak D., Cymbaluk-Płoska A., Bumbulienė Ž., Syrenicz A. (2024). Evaluation of trabecular bone microarchitecture and bone mineral density in young women, including selected hormonal parameters.. Biomedicines.

[31] Cheng C.H., Chen L.R., Chen K.H. (2022). Osteoporosis due to hormone imbalance: An overview of the effects of estrogen deficiency and glucocorticoid overuse on bone turnover.. Int. J. Mol. Sci..

[32] Delgado-Calle J., Bellido T. (2022). The osteocyte as a signaling cell.. Physiol. Rev..

[33] Han T.S., Cadge B., Conway G.S. (2006). Hearing impairment and low bone mineral density increase the risk of bone fractures in women with Turner’s syndrome.. Clin. Endocrinol. (Oxf.).

[34] Szybiak W., Kujawa B., Miedziaszczyk M., Lacka K. (2023). Effect of growth hormone and estrogen replacement therapy on bone mineral density in women with turner syndrome: A meta-analysis and systematic review.. Pharmaceuticals.

[35] Nour M.A., Burt L.A., Perry R.J., Stephure D.K., Hanley D.A., Boyd S.K. (2016). Impact of growth hormone on adult bone quality in turner syndrome: A HR-pQCT study.. Calcif. Tissue Int..

[36] Krugh M., Langaker M.D., Jan (2025). Dual-energy X-Ray absorptiometry. 2024 May 20. StatPearls [Internet]..

[37] Bakalov V.K., Chen M.L., Baron J., Hanton L.B., Reynolds J.C., Stratakis C.A., Axelrod L.E., Bondy C.A. (2003). Bone mineral density and fractures in Turner syndrome.. Am. J. Med..

[38] Silva B.C., Leslie W.D., Resch H., Lamy O., Lesnyak O., Binkley N., McCloskey E.V., Kanis J.A., Bilezikian J.P. (2014). Trabecular bone score: A noninvasive analytical method based upon the DXA image.. J. Bone Miner. Res..

[39] McCloskey E.V., Odén A., Harvey N.C., Leslie W.D., Hans D., Johansson H., Barkmann R., Boutroy S., Brown J., Chapurlat R., Elders P.J.M., Fujita Y., Glüer C.C., Goltzman D., Iki M., Karlsson M., Kindmark A., Kotowicz M., Kurumatani N., Kwok T., Lamy O., Leung J., Lippuner K., Ljunggren Ö., Lorentzon M., Mellström D., Merlijn T., Oei L., Ohlsson C., Pasco J.A., Rivadeneira F., Rosengren B., Sornay-Rendu E., Szulc P., Tamaki J., Kanis J.A. (2016). A meta-analysis of trabecular bone score in fracture risk prediction and its relationship to FRAX.. J. Bone Miner. Res..

[40] Leslie W.D., Krieg M.A., Hans D., Manitoba Bone Density Program (2013). Clinical factors associated with trabecular bone score.. J. Clin. Densitom..

[41] (2022). The jamovi project (2022).. https://www.jamovi.org.

[42] Itonaga T., Koga E., Nishigaki S., Kawai M., Sakakibara H., Hasegawa Y. (2020). A retrospective multicenter study of bone mineral density in adolescents and adults with Turner syndrome in Japan.. Endocr. J..

[43] Nguyen H.H., Wong P., Strauss B.J., Ebeling P.R., Milat F., Vincent A. (2018). A cross-sectional and longitudinal analysis of trabecular bone score in adults with turner syndrome.. J. Clin. Endocrinol. Metab..

[44] Hasegawa Y., Ikegawa K., Munenaga T., Itonaga T., Mitani-Konno M., Kawai M., Amano N. (2025). Bone health and pubertal induction in turner syndrome: the possibility of earlier transdermal lower-dose estradiol therapy for healthy bone density and quality.. Am. J. Med. Genet. C. Semin. Med. Genet..

[45] Mazzetti G., Berger C., Leslie W.D., Hans D., Langsetmo L., Hanley D.A., Kovacs C.S., Prior J.C., Kaiser S.M., Davison K.S., Josse R., Papaioannou A., Adachi J.R., Goltzman D., Morin S.N., CaMos Research Group (2017). Densitometer-specific differences in the correlation between body mass index and lumbar spine trabecular bone score.. J. Clin. Densitom..

[46] Palomo T., Dreyer P., Muszkat P., Weiler F.G., Bonansea T.C.P., Domingues F.C., Vieira J.G.H., Silva B.C., Brandão C.M.A. (2022). Effect of soft tissue noise on trabecular bone score in postmenopausal women with diabetes: A cross sectional study.. Bone.

[47] Palomo T., Muszkat P., Weiler F.G., Dreyer P., Brandão C.M.A., Silva B.C. (2022). Update on trabecular bone score.. Arch. Endocrinol. Metab..

[48] Kawai T., Autieri M.V., Scalia R. (2021). Adipose tissue inflammation and metabolic dysfunction in obesity.. Am. J. Physiol. Cell Physiol..

[49] Ali D., Tencerova M., Figeac F., Kassem M., Jafari A. (2022). The pathophysiology of osteoporosis in obesity and type 2 diabetes in aging women and men: The mechanisms and roles of increased bone marrow adiposity.. Front. Endocrinol. (Lausanne).

[50] Armutcu F., McCloskey E. (2025). Fracture risk assessment in metabolic syndrome in terms of secondary osteoporosis potential. A narrative review.. Calcif. Tissue Int..

[51] Jeong H.G., Park H. (2022). Metabolic disorders in menopause.. Metabolites.

[52] Davis S.M., Geffner M.E. (2019). Cardiometabolic health in Turner syndrome.. Am. J. Med. Genet. C. Semin. Med. Genet..

[53] Cameron-Pimblett A., La Rosa C., Davies M.C., Suntharalingham J.P., Ishida M., Achermann J.C., Conway G.S. (2025). Characterization of turner syndrome-associated diabetes mellitus.. J. Clin. Endocrinol. Metab..

[54] Hofbauer L.C., Busse B., Eastell R., Ferrari S., Frost M., Müller R., Burden A.M., Rivadeneira F., Napoli N., Rauner M. (2022). Bone fragility in diabetes: novel concepts and clinical implications.. Lancet Diabetes Endocrinol..

[55] Ho-Pham L.T., Nguyen T.V. (2019). Association between trabecular bone score and type 2 diabetes: a quantitative update of evidence.. Osteoporos. Int..

[56] Nardone I., Antonelli R., Zaccaria S., Wolde Sellasie S., Falcone S., Pecchioli C., Giurato L., Uccioli L. (2024). Prevalence of diabetes mellitus and clinical differences in patients with severe osteoporosis and fragility fractures.. J. Clin. Med..

[57] Napoli N., Strotmeyer E.S., Ensrud K.E., Sellmeyer D.E., Bauer D.C., Hoffman A.R., Dam T.T.L., Barrett-Connor E., Palermo L., Orwoll E.S., Cummings S.R., Black D.M., Schwartz A.V. (2014). Fracture risk in diabetic elderly men: The MrOS study.. Diabetologia.

[58] Napoli N., Chandran M., Pierroz D.D., Abrahamsen B., Schwartz A.V., Ferrari S.L., IOF Bone and Diabetes Working Group (2017). Mechanisms of diabetes mellitus-induced bone fragility.. Nat. Rev. Endocrinol..

[59] Dhaliwal R., Cibula D., Ghosh C., Weinstock R.S., Moses A.M. (2014). Bone quality assessment in type 2 diabetes mellitus.. Osteoporos. Int..

[60] Cameron- Pimblett A., La Rosa C., King T.F.J., Davies M.C., Conway G.S. (2017). The turner syndrome life course project: Karyotype- phenotype analyses across the lifespan.. Clin. Endocrinol. (Oxf.).

